# lncRNA ZEB1-AS1 promotes pulmonary fibrosis through ZEB1-mediated epithelial–mesenchymal transition by competitively binding miR-141-3p

**DOI:** 10.1038/s41419-019-1339-1

**Published:** 2019-02-12

**Authors:** Weibin Qian, Xinrui Cai, Qiuhai Qian, Wei Peng, Jie Yu, Xinying Zhang, Li Tian, Can Wang

**Affiliations:** 1 Department of Lung Disease, Affiliated Hospital of Shandong University of Traditional Chinese Medicine, Jinan, Shandong 250011 People’s Republic of China; 2 Department of Traditional Chinese Medicine, Shandong Academy of Occupational Health and Occupational Medicine, Shandong Academy of Medical Sciences, Jinan, Shandong 250062 People’s Republic of China; 3 Department of Endocrinology, Affiliated Hospital of Shandong University of Traditional Chinese Medicine, Jinan, Shandong 250011 People’s Republic of China; 4 Department of Scientific Research, Affiliated Hospital of Shandong University of Traditional Chinese Medicine, Jinan, Shandong 250011 People’s Republic of China; 5 Department of Chinese Internal Medicine, Shandong University of Traditional Chinese Medicine, Jinan, Shandong 250355 People’s Republic of China; 6 First Clinical Medical College, Shandong University of Traditional Chinese Medicine, Jinan, Shandong 250355 People’s Republic of China

## Abstract

Long non-coding RNAs (lncRNAs) have been reported to be involved in various pathophysiological processes in many diseases. However, the role and mechanism of lncRNAs in pulmonary fibrosis have not been explicitly delineated. In the present study, we found that lncRNA ZEB1 antisense RNA 1 (ZEB1-AS1) is upregulated in the lungs of BLM-induced rats and TGF-β1-induced RLE-6TN cells, and positively correlated with the levels of ZEB1, an epithelial–mesenchymal transition (EMT) master regulator. Knockdown of ZEB1-AS1 alleviated BLM-induced fibrogenesis, in vivo, via inhibiting EMT progress. Mechanistically, we identified that ZEB1-AS1 promoted fibrogenesis in RLE-6TN cells and ZEB1-AS1 silencing inhibited TGF-β1-induced fibrogenesis through modulation of miR-141-3p. Further experiments revealed that ZEB1-AS1 acted as competing endogenous RNA (ceRNA) of miR-141-3p: forced expression of ZEB1-AS1 reduced the expression of miR-141-3p to activate Zinc-finger Ebox Binding Homeobox 1 (ZEB1) in RLE-6TN cells. In addition, we found that upregulation of miR-141-3p prevented fibrogenesis by targeting ZEB1. Therefore, our finding suggested lncRNA ZEB1-AS1 as a new profibrotic molecule that acts as a regulator of miR-141-3p/ZEB1 axis during lung fibrosis and demonstrated ZEB1-AS1 as a potential therapeutic target for the prevention and treatment of pulmonary fibrosis.

## Introduction

Idiopathic pulmonary fibrosis (IPF) a chronic, progressive interstitial pneumonia with an unknown etiology. It is characterized by the patterns of usual interstitial pneumonia in radiologic and/or histopathologic manifestation^1–3^. Currently, plenty of mechanisms, inducing abnormal remodeling^4^, epithelial damage^5^, cell senescence^6,7^, and immune response^8^ are proposed as relevant in this complexity. Accumulation of activated fibroblasts/myofibroblasts and deposition of excessive extracellular matrix (ECM) are crucial processes for fibrotic remodeling in IPF^9^. However, the origin and process of activation of fibroblasts/myofibroblasts during fibrotic remodeling remain largely undefined.

Epithelial–mesenchymal transition (EMT) may be one of the mechanisms mediating the expansion of fibroblasts/myofibroblasts^10^. Transforming growth factor-β1 (TGF-β1), one of the major profibrotic cytokines in IPF, is considered to act as a master switch in EMT^11^. Following exposure to TGF-β1, alveolar epithelial cells undergo EMT as evidenced by decreased expression of epithelial markers (E-Cadherin), increased expression of mesenchymal markers (α-SMA, collagen 1 and fibronectin 1)^12–14^. In our previous studies, we used intratracheal administration of bleomycin (BLM) to successfully establish a rat model of pulmonary fibrosis, and found inhibition of TGF-β1-mediated EMT is sufficient to alleviate IPF in rats^15^. Therefore, inhibiting the development of EMT might serve as a potential target for IPF treatment.

Zinc-finger E-box binding homeobox 1 (ZEB1) is a transcription factor that promotes tumor invasion and metastasis by inducing EMT in carcinoma cells^16,17^. Recently, increased ZEB1 expression was detected in alveolar epithelium adjacent to sites of ECM deposition in IPF lung tissue^18^, suggesting that ZEB1-dependent EMT of alveolar cells contributes to fibrosis and ZEB1 could be a therapeutic target for the prevention of IPF. Intriguing, we noticed that long non-coding RNAs (lncRNAs) are recently identified novel regulator that could control ZEB1 expression. LncRNAs are defined as long RNA transcripts with no protein-coding capacity, which been found extensively involved in different biology progresses, including cell cycle, apoptosis, metabolism, and EMT. Wang et al.^19^ uncovered that HOTAIR mediates osteosarcoma progress by upregulating ZEB1 expression via acting as a competitive endogenous RNA (ceRNA) via miR-217. Enhanced expression of PTAR can promote EMT and metastasis of ovarian cancer through the regulation of miR-101/ZEB1 axis^20^. Meanwhile, the ceRNA role of lncRNA in IPF has also been noticed^21,22^. lncRNA ZEB1 antisense RNA 1 (ZEB1-AS1) gene is located in physical contiguity with ZEB1, a crucial transcription factor regulating EMT^23^. ZEB1-AS1 was discovered as an oncogene in a plenty of cancers by epigenetically activating ZEB1^24–27^. However, its expression profile and role in IPF remains unknown. We wondered if ZEB1-AS1 involves in pulmonary fibrosis and the potential role it may play.

In this study, we investigated the impact and mechanisms of ZEB1-AS1 on EMT involved in IPF. Our results showed that ZEB1-AS1 was significantly upregulated in IPF, and positively correlated with ZEB1 expression. ZEB1-AS1 promoted fibrogenesis by acting as a ceRNA for miR-141-3p in alveolar type II epithelial (RLE-6TN) cells. Moreover, knockdown of ZEB1-AS1 alleviated lung fibrosis by suppressing EMT progress in TGF-β1-induced RLE-6TN cells and in BLM-treatment rats. Overexpression of miR-141-3p retarded TGF-β1-induced EMT by targeting ZEB1. These findings indicate that ZEB1-AS1 is a potential EMT inducer and may be considered a new therapeutic target for IPF.

## Results

### ZEB1-AS1 was highly expressed in pulmonary fibrosis and correlated with ZEB1 expression

**Fig. 1:**
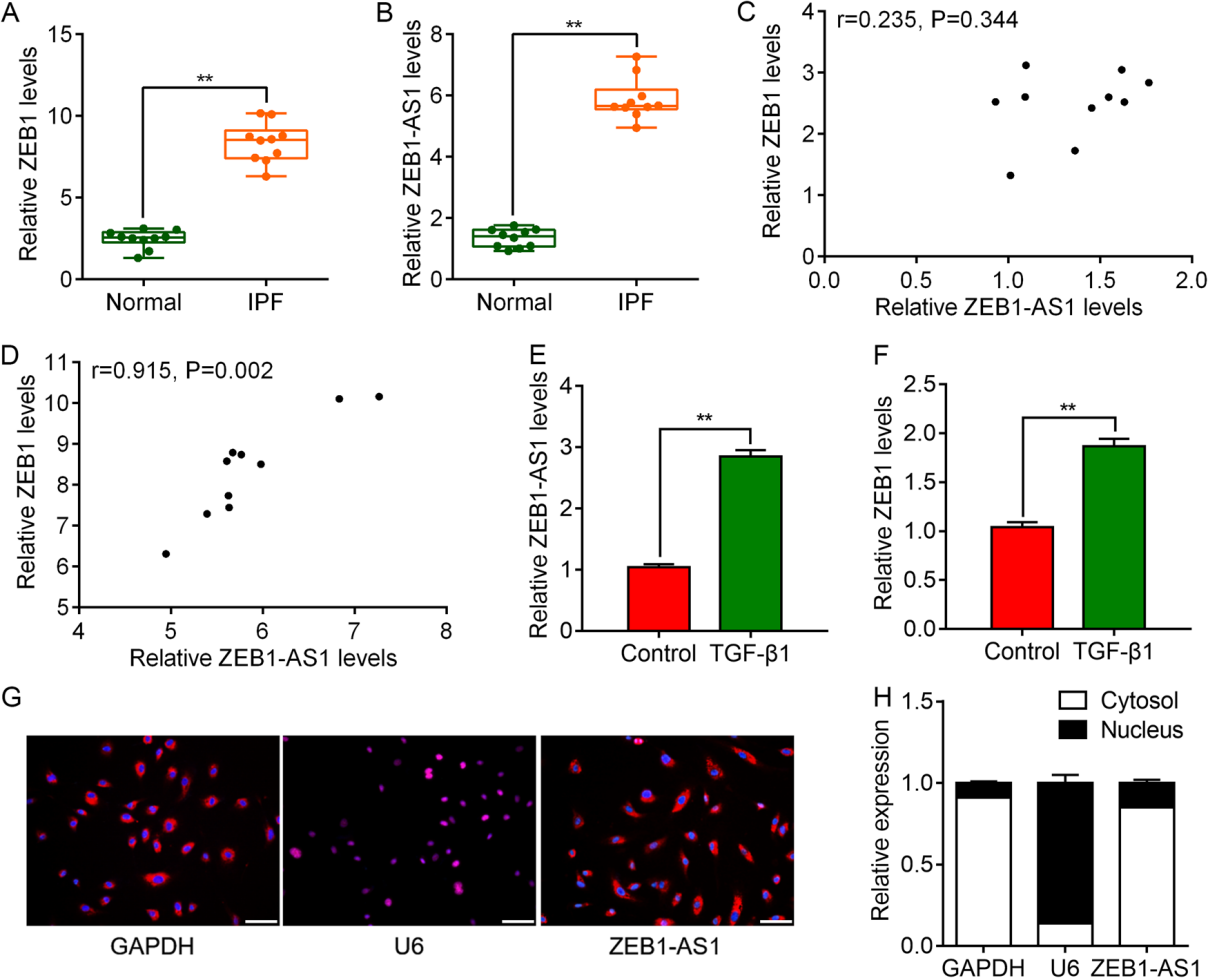
Upregulated ZEB1-AS1 in pulmonary fibrosis is positively correlated with ZEB1 expression. RT-qPCR was carried out to determine the relative expression of ZEB1-AS1 (**a**) and ZEB1 (**b**) mRNA in BLM-induced lung tissues (IPF group, *n* = 10) and normal lung tissues (Normal group, *n* = 10). Spearman analysis was used to analyze the association between ZEB1-AS1 and ZEB1 expression in the lung tissues from Normal group (**c**) and IPF group (**d**). Relative expression of ZEB1-AS1 (**e**) and ZEB1 (**f**) mRNA in RLE-6TN cells treated with 10 ng/ml TGF-β1 for 48 h, as detected by RT-qPCR. **g** RNA FISH was performed to determine the location of endogenous ZEB1-AS1 (red) in RLE-6TN cells. U6 and GAPDH were used as nuclear and cytoplasmic localization markers, respectively. DNA (blue) was stained with DAPI. **h** Nucleocytoplasmic separation result confirmed that ZEB1-AS1 was mainly expressed in the cytoplasm by using RT-qPCR. *P < 0.05, **P < 0.01

Firstly, we identified that the expression levels of ZEB1 mRNA (Fig. 1a) and lncRNA ZEB1-AS1 (Fig. 1b) were upregulated in BLM-induced rat model of IPF, compared with normal control. We found no linear relation between ZEB1-AS1 and ZEB1 expression levels in normal lung tissues (Fig. 1c). However, a positive correlation between the expression levels of ZEB1-AS1 and ZEB1 was observed (Fig. 1d). Moreover, TGF-β1 treatment for 48 h resulted in significantly increase in both ZEB1-AS1 (Fig. 1e) and ZEB1 (Fig. 1f) levels in RLE-6TN cells. Using fluorescent in situ hybridization (FISH) (Fig. 1g) and confirmed by quantifying nuclear/cytoplasmic RNA (Fig. 1h), we identified that ZEB1-AS1 transcripts were more localized in the cytoplasm than in the nucleus. Collectively, these results suggest that upregulated ZEB1-AS1 in pulmonary fibrosis is associated with ZEB1 expression, suggesting it may involve in the development of pulmonary fibrosis.

### Knockdown of ZEB1-AS1 alleviates pulmonary fibrosis, in vivo

**Fig. 2:**
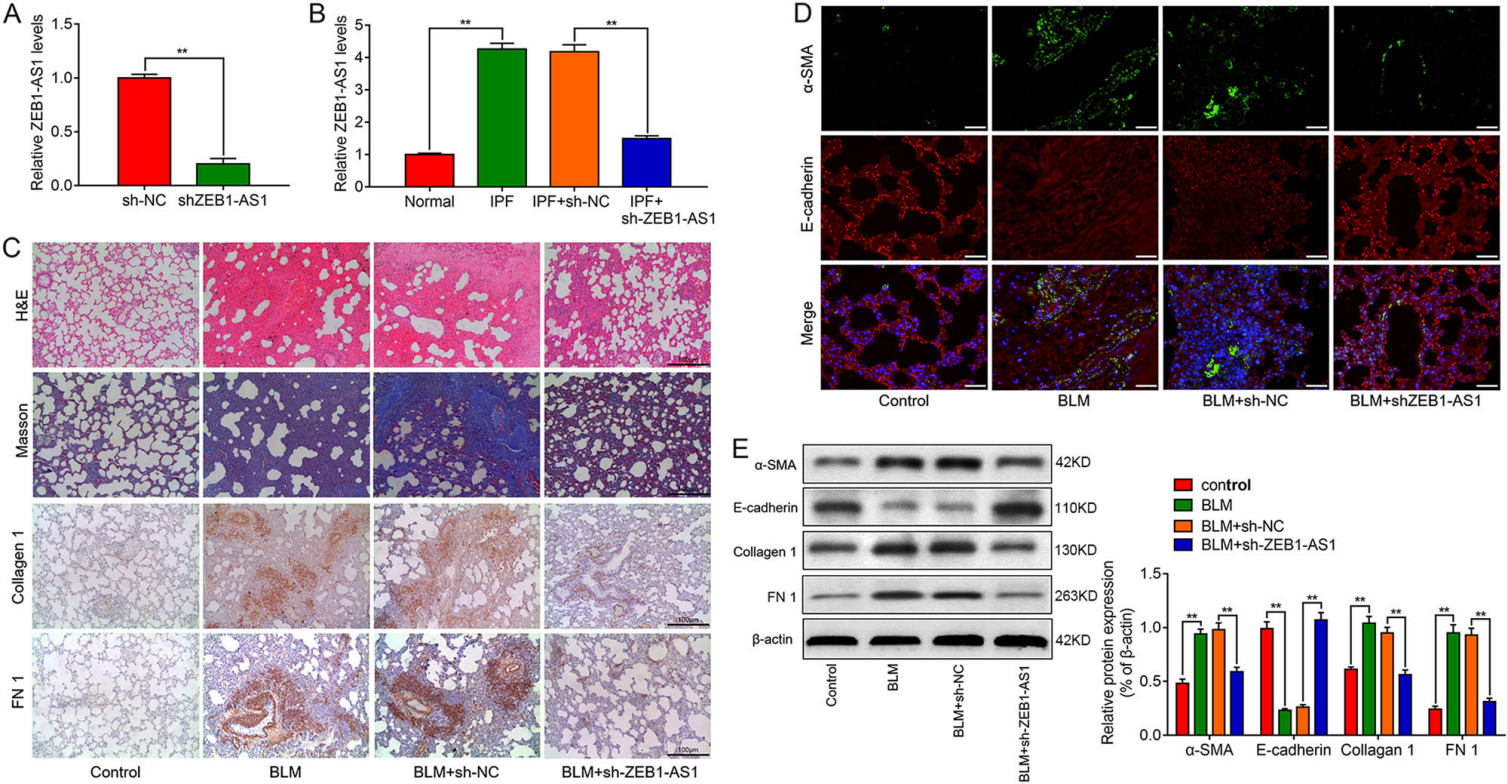
Knockdown of lncRNA ZEB1-AS1 inhibits bleomycin (BLM)-induced pulmonary fibrosis, in vivo. **a** RT-qPCR analysis showed the expression of ZEB1-AS1 in the lungs from normal rats and rats treated with adenovirus associated virus 5 (AAV5) carrying sh-ZEB1-AS1 (*n* = 10). **b** Treatment with AAV5 carrying sh-ZEB1-AS1 showed decreased ZEB1-AS1 expression compared with those in the BLM group as confirmed by RT-qPCR analysis (*n* = 10). **c** H&E and Masson’s trichrome staining indicated the collagen deposition, and the areas of fibrosis in rats with or without knockdown of ZEB1-AS1; IHC analysis showed the expression of levels of collagen 1 and fibronectin 1 (FN1) in lung tissues after knockdown of ZEB1-AS1. **d** Double-labeling immunofluorescence demonstrated the overlap of E-cadherin (red) and a-SMA (green) in lung tissues after knockdown of ZEB1-AS1. **e** Western blot analysis showed the relative expression of EMT-related markers, α-SMA, E-cadherin, Collagen 1, and FN1 protein were detected in indicated groups (means ± SD, *n* = 3). *P < 0.05, **P < 0.01

To exactly investigate the effect of ZEB1-AS1 in IPF, rats were administrated with adenovirus carrying shRNA against ZEB1-AS1 (sh-ZEB1-AS1) or a negative control (sh-NC) immediately after injection with BLM. We found that lung tissues treated with sh-ZEB1-AS1 displayed ZEB1-AS1 inhibition (Fig. 2a), and sh-ZEB1-AS1 treatment inhibited the upregulation of ZEB1-AS1 in BLM-treated rats (Fig. 2b). BLM-induced rat model of lung fibrosis was established as verified by H&E-staining and Masson’s trichrome assays for collagen deposition. However, knockdown of ZEB1-AS1 significantly alleviated pulmonary fibrosis comparable to the BLM group (Fig. 2c). Meanwhile, immunohistochemistry analysis illustrated that the levels of fibrosis-relevant protein collagen 1 and FN1 were increased in the lung tissues from BLM-treated mice compared with those in the control group (Fig. 2c). It has been reported that EMT plays a key role in pulmonary fibrosis^28^. Using double immunofluorescence, we found that the expression of E-cadherin protein was decreased, while the expression of α-SMA protein was increased after BLM injection, and above changes were reversed in the ZEB1-AS1 silenced lung tissues (Fig. 2d). In addition, western blot analysis showed the levels of collagen 1, FN1 and a-SMA protein were reduced, and E-cadherin protein was increased in lung tissues infected with sh-ZEB1-AS1 following BLM treatment (Fig. 2e). Taken together, above results indicated that knockdown of ZEB1-AS1 alleviated BLM-induced pulmonary fibrosis through inhibiting EMT progress.

### ZEB1-AS1 promotes fibrogenesis dependent on miR-141-3p

**Fig. 3:**
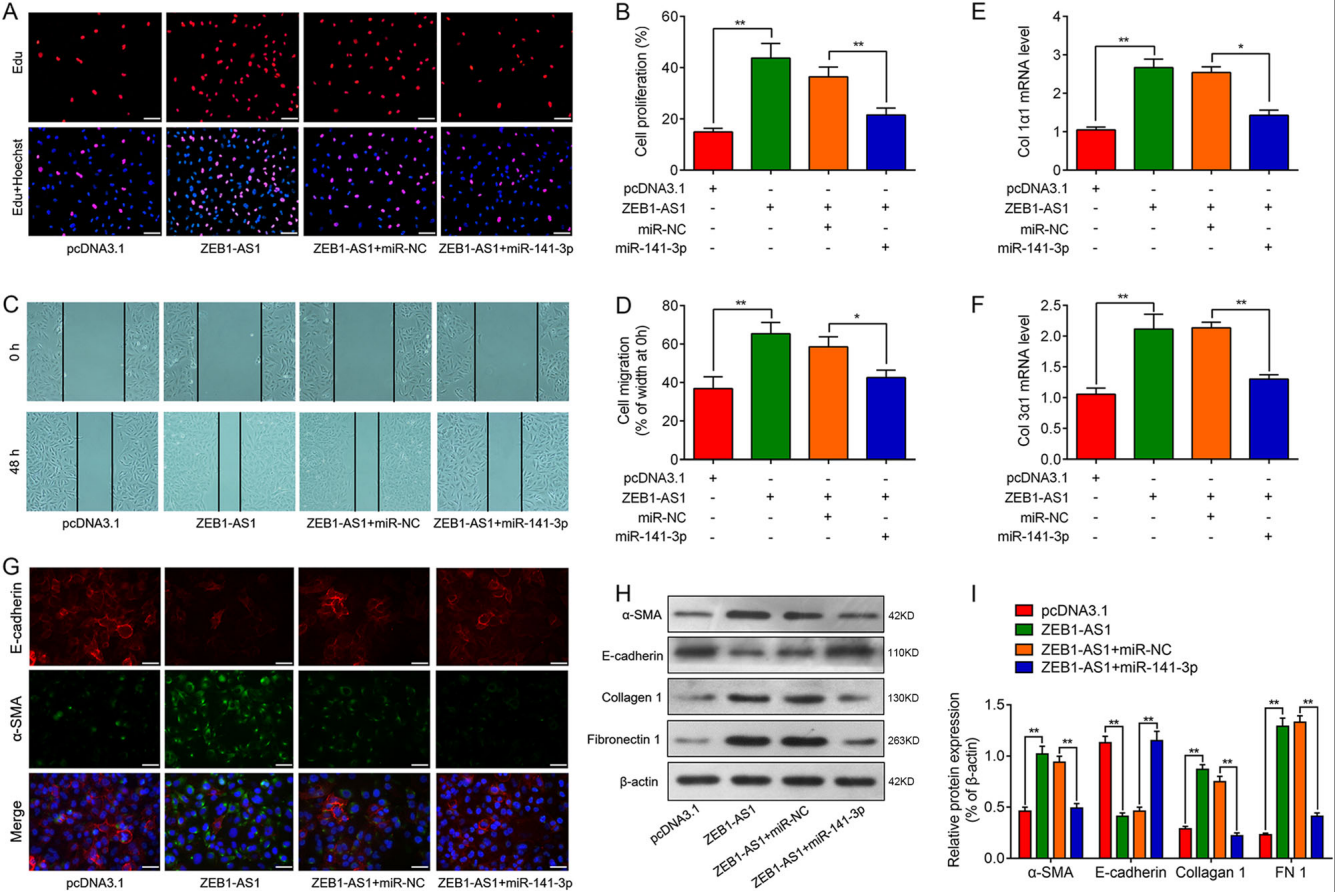
lncRNA ZEB1-AS1 contributes to fibrogenesis in alveolar type II epithelial cells by inhibiting the function of miR-141-3p. **a**, **b** EdU incorporation assay was used to assess the effect of ZEB1-AS1 on cells proliferation in RLE-6TN with or without upregulation of miR-141-3p. Scale bar = 100 μm. **c**, **d** Wound-healing assay reveals the effect of ZEB1-AS1 on cell migration in RLE-6TN cells with or without upregulation of miR-141-3p. Scale bar = 200 μm. RT-qPCR was performed to measure the expression of Col 1α1 (**e**) and Col 3α1 (**f**) in RLE-6TN cells following co-transfection of pcDNA3.1 or ZEB1-AS1 vector, miR-141-3p or miR-NC. **g** Representative images of immunofluorescence staining in each group to determine protein expression of E-cadherin (red) and a-SMA (green) accompanied with nuclei stained by DAPI (blue) in RLE-6TN cells in groups, as indicated. **h**, **i** Western blot analysis was performed to measure the expression of epithelial–mesenchymal transition (EMT)-related proteins α-SMA, E-cadherin, Collagen 1, and Fibronectin 1 (FN1), and β-actin was used as a loading control (means ± SD, *n* = 3). *P < 0.05, **P < 0.01

In order to determine the role of ZEB1-AS1 during the fibrogenesis, we examined effects of ZEB1-AS1 on the cell proliferation and migration in cultured rat alveolar type II cells (RLE-6TN cells). As shown in Fig. 3d, overexpression of ZEB1-AS1 promoted cells proliferation and migration, which was attenuated by overexpression of miR-141-3p. In addition, reverse transcription quantitative PCR (RT-qPCR) analysis showed that the overexpression of ZEB1-AS1 increased collagen 1α1 and collagen 3α1 mRNA levels, which was abrogated by the co-transfection of cells with miR-141-3p (Fig. 3e, f). Immunofluorescence demonstrated that overexpression of ZEB1-AS1 significantly increased the levels of α-SMA and decreased the expression of E-cadherin in RLE-6TN cells. However, overexpression of miR-141-3p could attenuate the above changes in α-SMA and E-cadherin (Fig. 3g). To quantificationally determine the role of ZEB1 during fibrogenesis, we performed western blot analysis and the results showed that enforced expression of ZEB1-AS1 induced fibroblast activation of RLE-6TN cells (as evidenced by the upregulated mesenchymal markers, including α-SMA, Collagen 1, FN 1, and loss of the epithelial cell marker E-cadherin) could be abolished after miR-141-3p overexpression (Fig. 3h, i).

**Fig. 4:**
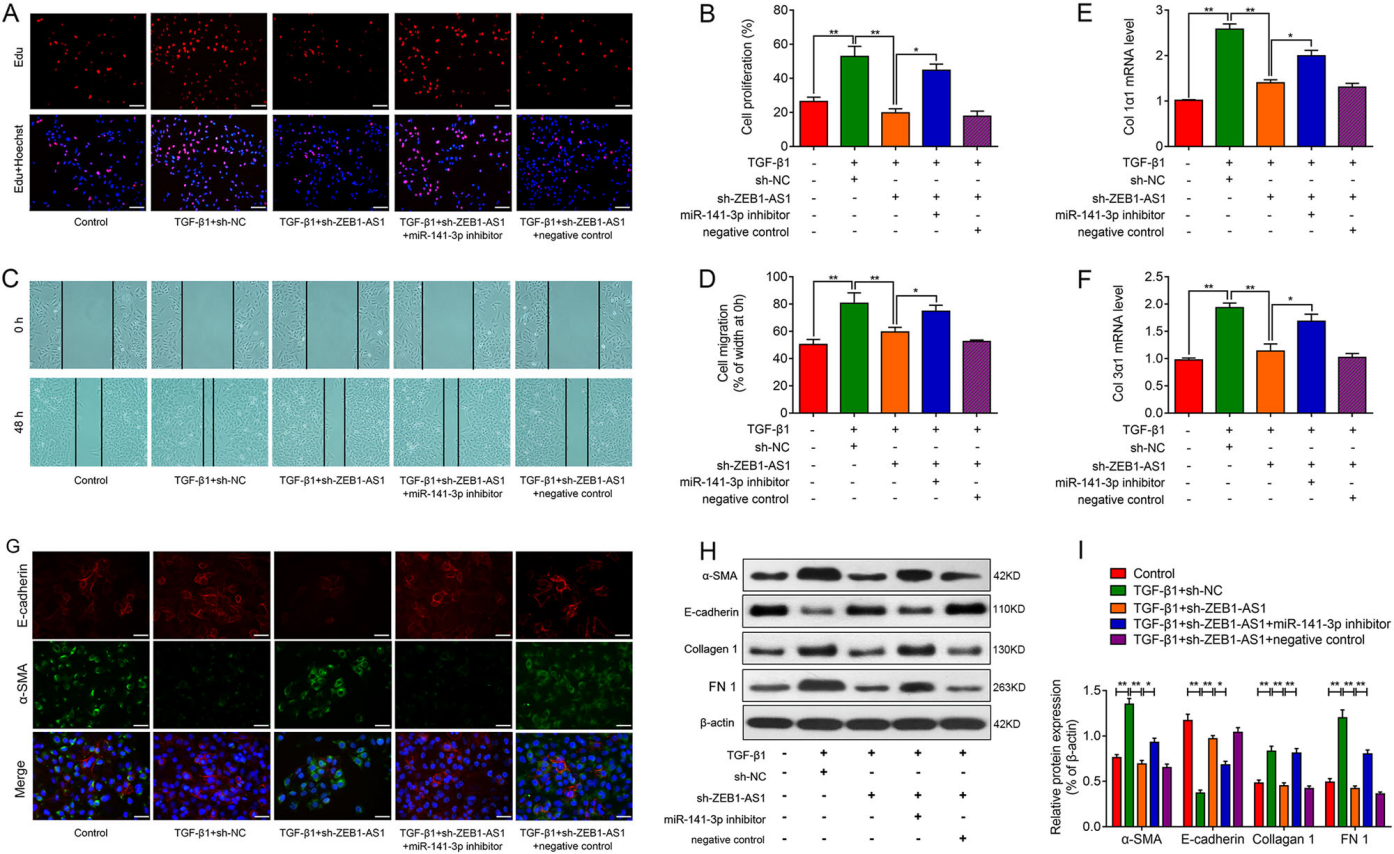
Knockdown of ZEB1-AS1 alleviates TGF-β1-induced fibrogenesis in alveolar type II epithelial cells through miR-141-3p regulation. **a**, **b** EdU incorporation assay was performed to assess the effect of ZEB1-AS1 depletion on cells proliferation in TGF-β1-induced RLE-6TN cells. Scale bar = 100 μm. **c**, **d** Wound-healing assay revealed the effect of ZEB1-AS1 migration ability in TGF-β1-induced RLE-6TN cells with or without downregulation of miR-141-3p. Scale bar = 200 μm. RT-qPCR was used to measure the expression of Col 1α1 (**e**) and Col 3α1 (**f**) mRNA in TGF-β1-treated RLE-6TN cells with ZEB1-AS1/miR-141-3p downregulation. **g** Double-labeling immunofluorescence demonstrated the protein expression of E-cadherin (red) and a-SMA (green) accompanied with nuclei stained by DAPI (blue) in RLE-6TN cells. **h**, **i** Western blot analysis showed the expression of epithelial–mesenchymal transition (EMT)-related proteins α-SMA, E-cadherin, Collagen 1, and Fibronectin 1 (FN1), and β-actin was used as a loading control (means ± SD, *n* = 3). *P < 0.05, **P < 0.01

To further validate the hypothesis that ZEB1-AS1 promotes TGF-β1-induced fibrogenesis, a loss-of-function approach was employed. As shown in Fig. 4a–d, knockdown of ZEB1-AS1 significantly alleviated TGF-β1-induecd proliferation and migration of RLE-6TN cells, as evidenced by Edu incorporation assay and wound-healing assay, respectively. However, suppression of miR-141-3p could abolish the effects of ZEB1-AS1 silencing on cell proliferation and migration in TGF-β1-treated RLE-6TN cells. As revealed in Fig. 4e, f, following treatment with the profibrotic cytokine TGF-β1 for 2 days, Col 1α1 and Col 3α1 mRNA were notably increased. More importantly, ZEB1-AS1 depletion downregulated TGF-β1-induced Col 1α1 and Col 3α1, which was abolished by the suppression of miR-141-3p. Subsequent immunofluorescence showed that ZEB1-AS1 knockdown overturned TGF-β1-induced loss of E-cadherin and production of α-SMA, whereas miR-141-3p knockdown reversed the above changes induced by ZEB1-AS1 depletion in RLE-6TN cells (Fig. 4g). Consistently, western blot analysis demonstrated that ZEB1-AS1 depletion alleviated the loss of E-cadherin and production of α-SMA, Collagen 1, FN 1 in RLE-6TN cells induced by TGF-β1, while miR-141-3p knockdown reversed the effects of ZEB1-AS1 silencing on the changes of EMT markers (Fig. 4h, i). Taken together, above results indicate that ZEB1-AS1 involves lung fibrosis through promoting EMT process by regulation of miR-141-3p.

### ZEB1-AS1 interacts with miR-141a-3p through directly binding to the 3ʹUTR

**Fig. 5:**
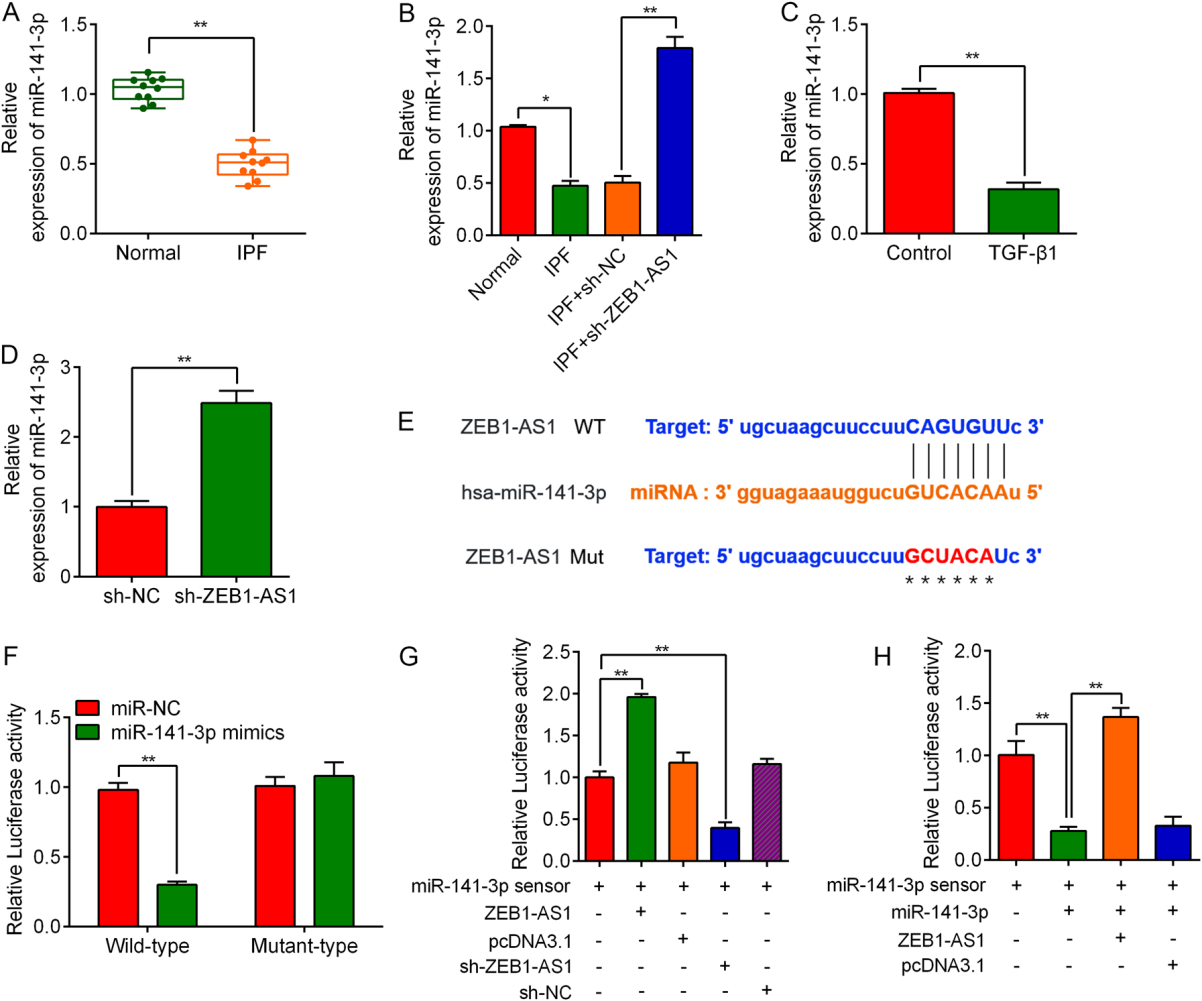
lncRNA ZEB1-AS1 regulates ZEB1 expression by sponging miR-141-3p. **a** RT-qPCR was used to determine the expression of miR-141-3p in BLM-induced lung tissues (IPF group, *n* = 10) and normal lung tissues (Normal group, *n* = 10). **b** Relative expression of miR-141-3p in the lung tissues with or without ZBE1-AS1 depletion. **c** Relative expression of miR-141-3p in RLE-6TN cells treated with 10 ng/ml TGF-β1 for 48 h. **d** Relative expression of miR-141-3p in RLE-6TN cells with or without knockdown of ZEB1-AS1. **e** Bioinformatics predicted the binding sites between ZEB1-AS1 and miR-141-3p and the schematic diagram shows the sequences of ZEB1-AS1 3ʹ-UTR wild-type and mutant with miR-141-3p. **f** The dual-luciferase reporter gene assay was performed to identify the interaction between ZEB1-AS1 and miR-141-3p. Luciferase activities were calculated as the ratio of firefly/renilla activities and normalized to the miR-NC + ZEB1-AS1 wile-type group. **g** ZEB1-AS1 decreased miR-141-3p activity. RLE-6TN cells were infected with the miR-141-3p sensor and then transfected with ZEB1-AS1 or sh-ZEB1-AS1. Luciferase activity of the miR-141-3p sensor was increased in cells treated with ZEB1-AS1, whereas decreased after knockdown of ZEB1-AS1. **h** ZEB1-AS1 acts as a sponge for miR-141-3p. RLE-6TN cells were infected with ZEB1-AS1 and pcDNA3.1 followed by transfection with the miR-141-3p sensor. ZEB1-AS1 ablated the inhibitory effects of miR-141-3p on its sensor, as determined by the luciferase assay. *P < 0.05, **P < 0.01

To further explore the mechanism whereby ZEB1-AS1 regulates the IPF progress, we focus on miR-141a-3p by using starBase V3.0, predicting miR-141a-3p as a potential target of ZEB1-AS1. miR-141a-3p expression was downregulated in IPF lung tissues (Fig. 5a) and knockdown of ZEB1-AS1 markedly increased miR-141a-3p expression (Fig. 5b). Consistently, TGF-β1 treatment for 48 h downregulated miR-141-3p expression in RLE-6TN cells (Fig. 5c) and ZEB1-AS1 silencing increased miR-141-3p expression, in vitro (Fig. 5d). To further confirm the relation of ZEB1-AS1 and miR-141-3p, a WT-ZEB1-AS1 3ʹUTR luciferase reporter vector, and a Mut-ZEB1-AS1 3ʹUTR luciferase reporter vector with mutations on miR-141-3p-binding site of the ZEB1-AS1 3ʹUTR was constructed (Fig. 5e). When co-transfected with miR-141-3p mimics, the luciferase activity of the WT-ZEB1-AS1 vector was notably suppressed, while no significant change with the luciferase activity of the Mutant type. was observed, compared with control group (Fig. 5f). The luciferase activity of the miR-141-3p sensor was increased in ZEB1-AS1 overexpressed RLE-6TN cells, suggesting that ZEB1-AS1 bound miR-141-3p to limit the inhibitory effect of the latter on luciferase activity. On the contrary, ZEB1-AS1 silencing significantly inhibited the luciferase activity of the miR-141-3p (Fig. 5g). Moreover, overexpression of ZEB1-AS1 alleviated the inhibitory effect of miR-141-3p on its sensor (Fig. 5h). These data indicated that ZEB1-AS1 inversely interacted with miR-141-3p through directly binding to the 3ʹUTR.

### ZEB1 is a direct target of miR-141a-3p

**Fig. 6:**
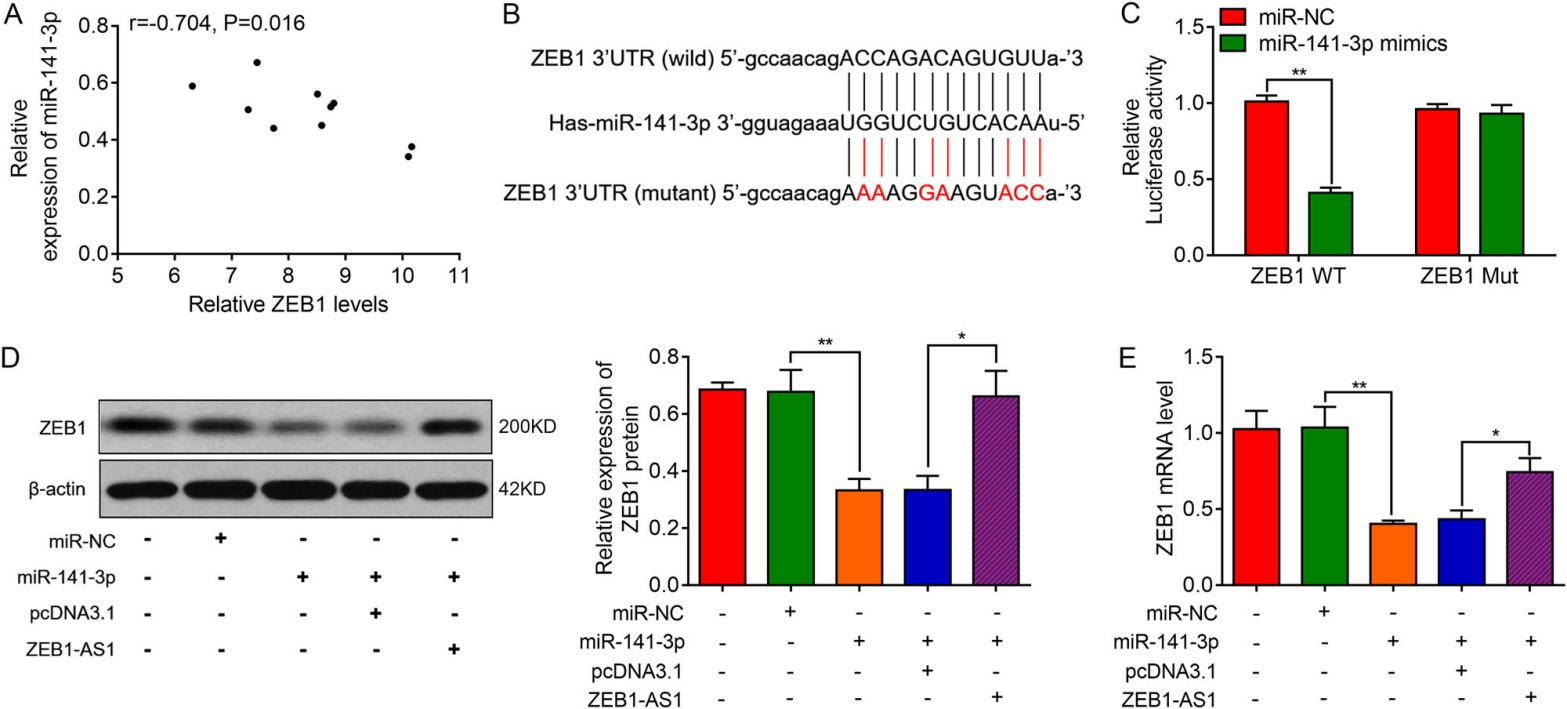
ZEB1-AS1/miR-141-3p downregulates the expression of ZEB1 in cultured alveolar type II epithelial cells. **a** Spearman analysis was used to analyze the association between miR-141-3p and ZEB1 expression; *r* = −0.704, *P* = 0.016. **b** The seed sequences of miR-141-3p match the 5ʹUTR of ZEB1, as predicted by starBase v3.0 (http://starbase.sysu.edu.cn/). **c** The 293T cells were co-transfected with 100 nM miR-141-3p or miRNA negative control (miR-NC) and the luciferase constructs carrying ZEB1 5ʹUTR. Luciferase assay was performed 24 h after transfection. **d** Western blot analysis and **e** RT-qPCR were performed to measure the expression of ZEB1 in ZEB1-AS1/miR-141-3p upregulated groups. *P < 0.05, **P < 0.01

From the experiments above, we can draw the conclusion that miR-141a-3p plays a pivotal role in the process of EMT, thus limiting fibrogenesis. We further identified a negative relationship between miR-141-3p and ZEB1 expression from the BLM-stimulated lung tissues (Fig. 6a). But, whether miR-141a-3p exerts its EMT-suppressive effects through regulation ZEB1 is still unknown. It was found that the ZEB1 3ʹUTR contained a complementary base sequence for miR-141-3p (Fig. 6b). Subsequent luciferase reporter assay showed that miR-141-3p restrained the luciferase activity of ZEB1-WT luciferase vector, yet there was no specific correlation between miR-141-3p and ZEB1-Mut vector (Fig. 6c). Moreover, we found upregulation of miR-141-3p could downregulate ZEB1 expression at both protein and mRNA levels and ZEB1-AS1 could reverse the inhibitory effects of miR-141-3p. These results strongly suggested that ZEB1 can be regulated by miR-141-3p directly.

### MiR-141a-3p blocks TGF-β1-induced EMT via targeting ZEB1

**Fig. 7:**
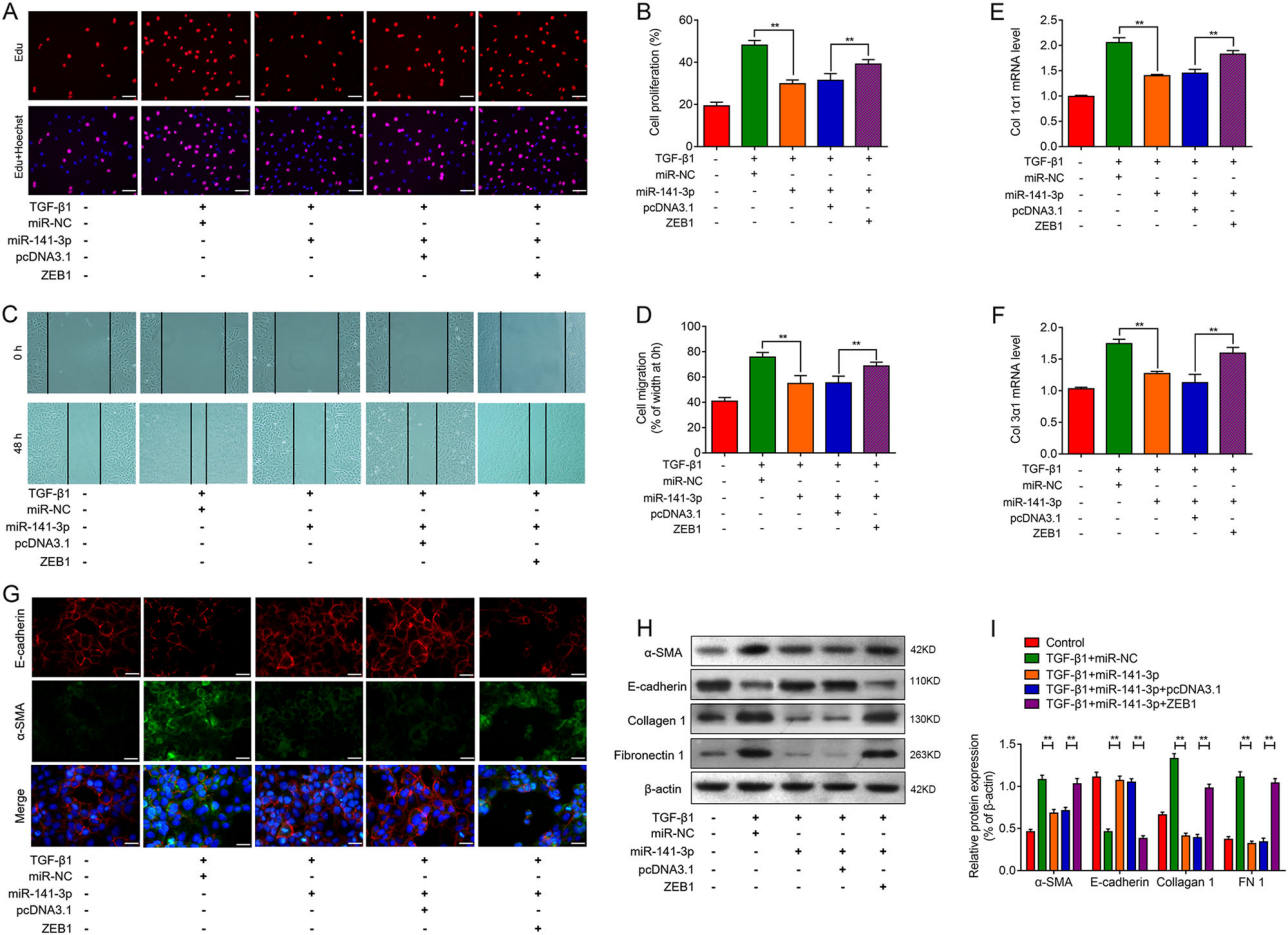
Overexpression of miR-141-3p alleviates TGF-β1-driven fibrogenesis in alveolar type II epithelial cells by targeting ZEB1. **a**, **b** EdU incorporation assay was used to assess the effect of miR-41-3p cells proliferation in TGF-β1-induced RLE-6TN cells. Scale bar = 100 μm. **c**, **d** Wound-healing assay reveals the effect of miR-41-3p on cell migration in TGF-β1-induced RLE-6TN cells. Scale bar = 200 μm. RT-qPCR was performed to detect the expression of Col 1α1 (**e**) and Col 3α1 (**f**) mRNA in TGF-β1-treated RLE-6TN cells following co-transfection of miR-141-3p, miR-NC, pcDNA3.1, or ZEB1 vector. **g** Double-labeling immunofluorescence demonstrated the overlap of E-cadherin (red) and a-SMA (green) in RLE-6TN cells in groups, described above. **h**, **i** Relative expression of EMT-related markers, α-SMA, E-cadherin, Collagen 1, and FN1 protein were detected in indicated groups through western blot analysis (means ± SD, *n* = 3). *P < 0.05, **P < 0.01

To further explore if miR-141-3p is required for fibrogenesis, RLE-6TN cells were transfected with either miR-141-3p mimics or control mimics (miR-NC) for 24 h and then treated with TGF-β1 for another 48 h. As illustrated in Fig. 7a–d, upregulation of miR-141-3p significantly inhibited TGF-β1-induecd proliferation and migration, which could be reversed by overexpression of ZEB1. More importantly, upregulation of miR-141-3p markedly attenuated the TGF-β1-induced Col 1α1 and Col 3α1 in RLE-6TN cells. In contrast, overexpression of ZEB1 blocked the effects of miR-141-3p on TGF-β1-induced Col 1α1 and Col 3α1 upregulation (Fig. 7e, f). Next, immunofluorescence results showed that upregulation of miR-141-3p suppressed the TGF-β1-induced EMT, whereas this effect was effectively reversed with the overexpression of ZEB1 (Fig. 7g). We also found that upregulation of miR-141-3p in RLE-6TN cells decreased TGF-β1-induced α-SMA, Collagen 1, and FN1 expression levels, and increased E-cadherin expression levels, when compared with control group. In contrast, overexpression of ZEB1 could at least partially restore the hallmarks of the EMT (Fig. 7i). These results indicate that miR-141-3p can inhibit TGF-β1-induced EMT by targeting ZEB1 in RLE-6TN cells.

## Discussion

In this study, to our knowledge, we for the first time showed that ZEB1-AS1 silencing inhibited BLM-induced pulmonary fibrosis by suppressing EMT progress. Mechanically, we identified that ZEB1-AS1 promotes pulmonary fibrosis by regulating ZEB1 expression and inducting EMT of alveolar type II epithelial cells by competitively binding miR-141-3p. Further studies showed that ZEB1, a key positive regulatory factor in EMT, as a direct target of miR-141-3p. Moreover, miR-141-3p could be suppressed by TGF-β1, and upregulation of miR-141-3p inhibited EMT by targeting ZEB1. Our results indicate that ZEB1-AS1 depletion may be a novel strategy for the prevention and treatment of IPF.

A growing body of evidence suggests that ZEB1 is involved in the process of organ fibrosis^29–31^. Park et al.^32^ identified that ZEB1 expression in pulmonary fibrosis patients significantly correlated with the fibrosis score and lung function decline, indicating that it may be related to the prognosis of pulmonary fibrosis. Importantly, Chilosi et al.^33^ suggested that the abnormal expression and localization of ZEB1 in bronchiolar fibro-proliferative lesions are unique for IPF, and might represent a specific marker in challenging lung biopsies. Considering the fact that inhibition of ZEB1 is able to protect against the development of renal^29,34^ and lung fibrosis^18^, and lncRNA ZEB1-AS1 can positively regulate the expression of ZEB1 and ZEB1 downstream molecules^27^, we hypothesize that ZEB1-AS1 may involve in the development of IPF by regulation of ZEB1. Aberrant lncRNA ZEB1-AS1 expression has been reported in many cancers^35–37^. It could and positively regulate ZEB1 expression, thus contributing to the initiation or progression of tumors. However, no study focused on the role of ZEB1-AS1 in IPF. Here, we firstly found that ZEB1-AS1 was highly expressed in lung tissues of IPF and TGF-β1-stimilated RLE-6TN cells. ZEB1-AS1 was correlated with ZEB1 expression in pulmonary lung tissues. Using bleomycin (BLM) to induce the animal model of IPF in rats, we found that knockdown of ZEB1-AS1 prevented pulmonary fibrosis by reducing ECM accumulation and EMT of alveolar epithelial cells. Therefore, further experiments were conducted to reveal the mechanisms underlying the action of ZEB1-AS1 in IPF.

Many studies have reported the correlation between lncRNAs and microRNAs (miRNAs) in IPF^22,38,39^. lncRNAs can participate in the ceRNA regulation network and regulate the functions, translational control and protein stability control of miRNAs^40,41^. Also, accumulating evidence has suggested that ZEB1 can be regulated by miRNAs. Liu et al.^24^ suggested that ZEB1-AS1 acts as an oncogene in osteosarcoma by epigenetically activating ZEB1. Similarly, another research demonstrated that ZEB1-AS1 functions as a molecular sponge for miR-200s and relieves the inhibition of ZEB1 caused by miR-200s in osteosarcoma^42^. Xiong et al.^25^ revealed a novel regulatory mechanism between ZEB1-AS1 and miR-101/ZEB1 axis in colorectal cancer. In the present study, we identified that ZEB1-AS1 promotes fibrogenesis by regulation of miR-141-3p. More importantly, using biological analysis and luciferase reporter assay, ZEB1-AS1 was confirmed as a target of miR-141-3p, in our study. MiR-141-3p is a member of miR-200 family that associated with the regulation of EMT and mesenchymal–epithelial transition^43^. Huang et al.^44^ found that upregulation of miR-141-3p hindered EMT by enhancing E-cadherin and decreasing vimentin expression in tubular epithelial with TGF-β1 treatment. However, to date, the role of miR-141-3p in pulmonary fibrosis is unclear. The results in our study showed that miR-141-3p was significantly downregulated in lung tissues of IPF and in TGF-β1-stimulated alveolar type II epithelial cells. More importantly, a recent study found knockdown of miR-141-3p upregulated ZEB1 expression and decreased E-cadherin expression in hepatocellular carcinoma cells^45^. Consistently, we showed that miR-141-3p inhibited the expression of ZEB1 in RLE-6TN cells in a transcriptional manner and upregulation of miR-141-3p blocks TGF-β1-induced EMT by directly targeting ZEB1. These results suggest that miRNA-141-3p plays a key role in ZEB1-AS1-induced EMT and based on the above results, it may come to the conclusion that suggested that ZEB1-AS1 moderates TGF-β1-induced EMT through regulation of ZEB1 via miR-141-3p sponging in IPF.

In conclusion, our integrated approach demonstrates that lncRNA ZEB1-AS1 promotes fibroblast activation and pulmonary fibrosis, by acting as a ceRNA for miR-141-3p to increase expression of ZBE1. Our results suggest that ZEB1-AS1/miR-141-3p/ZEB1 axis as an important player in TGF-β1-mediated EMT of alveolar type II epithelial cells and may provide new insights into the mechanisms underlying IPF.

## Materials and methods

### Pulmonary fibrosis model and treatment

Sprague-Dawley rats (180–220 g) were used as animal models. The rats were fed with conventional laboratory diet and free access to water in an air-conditioned room at approximately 25 °C. The rats were administered with BLM (Nippon Kayaku, Tokyo, Japan) intratracheally at a dose of 5 mg/kg dissolved in a total of 0.1 ml of sterile saline. The control groups were treated with 0.05 ml of sterile saline using the same method. The interfered sequence of ZEB1-AS1 was packaged in the adenovirus. The treatment of adenovirus packaging interfered with the sequence of ZEB1-AS1 was as follows: 40 rats were randomly divided into four groups (ten per group): control, BLM group, BLM + empty vector, and BLM + sh-ZEB1-AS1. The adenovirus associated virus 5 (AAV5) packaging interfered with ZEB1-AS1 sequence was sprayed into the rat lung tissues by using a Penn-Century MicroSprayer (Penn-Century Inc., PA, USA). All rats were sacrificed with overdose chloral hydrate at the 28th day after treatment. Lung tissue sections were collected and immediately frozen in liquid nitrogen for further studies. All animal procedures were conducted in accordance with humane animal care standards approved by the Ethics Committee of Affiliated Hospital of Shandong University of Traditional Chinese Medicine.

### Pathological staining

The pulmonary tissues were immersed in 4% paraformaldehyde for 24 h and transferred to 70% ethanol. After dehydrated, the samples were embedded in paraffin. and sectioned at 5-μm thickness. Sections were stained with hematoxylin and eosin (H&E) or Masson’s trichrome kit (Nanjing Jiancheng Co., Ltd., China) in according with the manufacturers’ instructions. Immunohistochemistry was performed to measure collagen 1 or fibronectin 1 (FN1) expression in lung tissues. After heat retrieval with heated citrate solution, endogenous peroxidase was blocked by incubation in 0.3% H_2_O_2_ for 10 min. Sections were incubated with primary antibody against collagen 1 (1:500, Abcam) or anti-fibronectin 1 (1:400, Abcam) overnight at 4 °C. Negative controls were performed by omitting the primary antibody. Subsequently, sections were incubated with Goat anti-rabbit HRP labeled secondary antibody (1:1000) for 30 min at room temperature, and developed with 3’3’-diaminobenzidine (DAB, DAKO) at a 1:50 concentration for 10 min. Hematoxylin was used for nuclear staining and images were acquired under a light microscope (Leica DM3000, Leica Company, Germany).

### Cell culture and treatment

Alveolar type II epithelial (RLE-6TN) cells was purchased form American Type Culture Collection (ATCC, Rockville, Maryland, USA). 293T cells was obtained from Cell Bank of Chinese Academy of Sciences (Shanghai, China). RLE-6TN cells were maintained in Ham’s F12 medium (Biowset, Riverside, MO, USA) supplemented with 10% Fetal Bovine Serum (FBS) (Gibco, MD, USA). 293T cells were cultured with RPMI 1640 medium (Invitrogen) supplemented with 10% FBS. All cells were cultured at 37 °C with 5% CO_2_ in humidified incubator. To establish the cell model of EMT, RLE-6TN cells were maintained in growth media added with 10 ng/ml TGF-β1 (R&D Systems, Minneapolis, MN) for 48 h, as previously described^15^.

### RNA transfection

The miR-141-3p inhibitor, miR-141-3p mimics, and homologous negative control were obtained from GenePharma (Shanghai, China). RLE-6TN cells were transfected above oligonucleotides using Lipofectamine 2000 reagent (Invitrogen) at the final concentration of 50–100 mM following the manufacturer’s instructions. Lentiviral short hairpin RNA (shRNA) vector as well as shRNA negative control (sh-NC) vector were commercially serviced by GeneChem Inc. (Shanghai, China). The shRNA targeting ZEB1-AS1 was 5ʹ-CCGGGCGCTCCTGTTTATGTACTTACTCGAGTAAGTACAT AAACAGGAGCGCTTTTTTG-3ʹ. RLE-6TN cells were transfected with sh-NC and sh-ZEB1-AS1 [multiplicity of infection = 100] diluted by Enhanced Infection Solution (ENi.S, pH 7.4). RT-qPCR was used to validate the transfection efficiency.

### Plasmid transfection

The complementary sequence of ZEB1-AS1 or ZEB1 was PCR-amplified and ligated into pcDNA3.1 ( + ) vector (Invitrogen, US) to construct ZEB1-AS1 or ZEB1 plasmid. The primers used were: ZEB1-AS1 forward 5ʹ- CCAGACACCTACACAACTTCC-3ʹ; reverse 5ʹ- GTGATCTCTACCCTCTTGCTT-3ʹ; ZEB1 forward 5ʹ-GCAGAUACUACACCAACUCTT-3ʹ; reverse 5ʹ-AUGGUGGUUAGUCAGUUGCTT-3ʹ. Transfections were performed using opti-MEM and lipofectamine 2000 reagents (Invitrogen) according to the manufacturer’s instructions and the efficiency was validated by RT-qPCR analysis.

### Immunofluorescence staining

Approximately 1 × 10^5^ RLE-6TN cells were cultivated on the sterile slides in a 24-well plate. After small RNA transfection and TGF-β1 treatment, these cells were rinsed with PBS for three times and fixed in 4% paraformaldehyde for 15 min. The cells were rinsed with PBS for three times, incubated with 0.5% Triton X-100 for 15 min at room temperature, and blocked with 4% BSA at 37 °C for 30 min. Afterward, the cells were incubated with the anti-E-cadherin antibody (1:500) and α-SMA antibody (1:400) at 4 °C overnight. Cells were then incubated with Alexa 594 Goat Anti-rabbit IgG2A or Alexa 488 Goat Anti-rabbit IgG at 37 °C for 1 h in dark. After rinsing with PBS for three times, the nuclei were stained with DAPI (Life Technologies Corporation) for 5 min at room temperature. Immunofluorescence was analyzed under a Nikon Eclipse 800 epifluorescence microscope with the appropriate filters.

### Fluorescence in situ hybridization (FISH)

RNA FISH was carried out to detect ZEB1-AS1 expression in RLE-6TN cells. After fixed with 4% formaldehyde for 15 min, cells were incubated with 0.2 mol/l HCl for 30 min, followed by 5 μg/ml proteinase K for 10 min. After acetylation in specific solution, hybridization reactions were performed using specific FITC-labeled ZEB1-AS1 probes (5 μg/ml) for 24 h in hybridization buffer at 55 °C. Cells were then washed twice with 2 × SSC wash buffer containing 0.01% Tween-20. Afterwards, FITC-labeled probes were detected using standard immunofluorescence protocols.

### RNA extraction and real-time PCR (RT-qPCR)

RNA was isolated from the tissue and cells by TRIzol reagent (Invitrogen). Complementary DNA (cDNA) was reversely transcribed using a PrimeScript reagent Kit With gDNA Eraser (Takara). The RT-qPCR was carried out with a SYBR Green PCR kit (TaKaRa) on an iCyler iQ Real-Time PCR System (Bio-Rad Laboratories Inc., USA). GAPDH was used as an endogenous control for detection of ZEB1-AS1, ZEB1, Col 1α1, and Col 3α1 mRNA. For quantitative miRNA analysis, total RNA was isolated using miRNeasy Mini kit (Qiagen). Mature miRNAs were reverse-transcripted with the Bulge-LoopTM miRNA qRT-PCR Starter Kit (Ribibio) in according with the manufacturer’s instructions. MiR-141-3p expression was measured by RT-qPCR and normalized to U6 small nuclear RNA. The reaction conditions were: 94 °C for 2 min, followed by 40 cycles at 94 °C for 10 s, 60 °C for 1 min, and 30 s at 72 °C. The primers for RT-qPCR were shown in Table 1. The fold change was calculated by the 2^−ΔΔCt^ method.

**Table 1:** The forward and reverse primers for real-time PCR

Genes		5ʹ–3ʹ primer sequence
ZEB1-AS1	Forward	TCCCTGCTAAGCTTCCTTCAGTGT
	Reverse	GACAGTGATCACTTTCATATCC
miR-141-3p	RT primer	GTTGGCTCTGGTGCAGGGTCCGAGGTATTCGCACCAGAGCCAACGATGTG
	Forward	CACATCCACCTCCTCCACATC
	Reverse	AATGCGGCCGCAACTCAATCAACATCACCAT
ZEB1	Forward	CGCAGTCTGGGTGTAATCGTAA
	Reverse	GACTGCCTGGTGATGCTGAAA
U6	Forward	GCTTCGGCAGCACATATACT
	Reverse	GTGCAGGGTCCGAGGTATTC
GAPDH	Forward	GGAGCGAGATCCCTCCAAAAT
	Reverse	GGCTGTTGTCATACTTCTCATGG

### Reporter constructs and luciferase reporter assay

Putative wild-type (WT) and mutant (Mut) miR-141-3p-binding sites in the 3ʹ-UTR of ZEB1-AS1 or ZEB1 mRNA, termed ZEB1-AS1-WT or ZEB1-AS1-Mut, and ZEB1-WT or ZEB1-Mut, were cloned into a pmirGLO-Report luciferase vector (Genearray Biotechnology, China). The reporter plasmid was transiently transfected into RLE-6TN cells in the presence of either miR-141-3p mimics and/or ZEB1-AS1. A miR-141-3p sensor reporter was constructed according to the method described previously^46^. As an internal control, 5 ng/well Renilla luciferase plasmid was used. Luciferase activity was evaluated with the Dual-Luciferase Reporter Assay System (Promega, WI, USA) 48 h after transfection.

### Western blot analysis

Total proteins were extracted tissues or cells using 1% radio immunoprecipitation assay (RIPA) buffer included proteinase inhibitor (Sigma-Aldrich; St. Louis, MO, USA). The quantity of protein in the lysates was measured using a BCA kit (Beyotime, Jiangsu, China), and equal amounts of proteins were separated by 12% sodium dodecyl sulfate polyacrylamide gel electrophoresis and transferred to Polyvinylidene fluoride (PVDF) membranes (Millipore). The membranes were then blocked with 5% skim milk for 1 h at room temperature, incubated with primary antibodies against α-SMA (ab5694, 1:1000, Abcam), E-cadherin (ab40772, 1:1000, Abcam), Collagen 1 (ab34710, 1:1000, Abcam), Fibronectin 1 (FN1, ab2413, 1:500, Abcam), ZEB1 (sc-515797, 1:1000, Santa Cruz), and β-actin (sc-70319, 1:1000, Santa Cruz) overnight at 4 °C. Subsequently, the blots were incubated with Goat Anti-Rabbit IgG H&L (HRP) (AS09 602, 1:10000, Agrisera) at room temperature for 1 h. Immunoblots were visualized by an enhanced chemiluminescence (ECL) detection system (Pierce, Rockford, IL) and the intensities of the signals were quantified using ImageJ software v1.8.0 (National Institutes of Health).

### Statistical analysis

All experiments were repeated at least thrice independently. All data are shown as mean ± standard deviation. Student’s *t*-test and one-way ANOVA with Student–Newman–Keuls post hoc test were employed to determine significance on SPSS 20.0 software (SPSS, Inc., Chicago, IL, USA). *P* < 0.05 was considered statistically significant.
